# Supplementary material for: Improvement of RNA In Situ Hybridisation for Grapevine Fruits and Ovules
Source: Int J Mol Sci. 2023 Jan 2;24(1):800. doi: 10.3390/ijms24010800 (PMC9821503; doi:10.3390/ijms24010800)
Supplement: Supplementary file 1 [file ijms-24-00800-s001.zip › ijms-2066766-supplementary.pdf]

Improvement of RNA in situ hybridization for grapevine fruit and ovule

Jin Yao[1, 2†], Xingmei Li[1, 2†], Na Wu[1, 2], Songlin Zhang[1,2], Min Gao[1,2*], Xiping Wang[1,2*]

1 State Key Laboratory of Crop Stress Biology in Arid Areas, College of Horticulture, Northwest A&F University, Yangling, Shaanxi 712100, China

2 Key Laboratory of Horticultural Plant Biology and Germplasm Innovation in Northwest China, Ministry of Agriculture, Northwest A&F University, Yangling, Shaanxi 712100, China

* Correspondence: mingao@nwafu.edu.cn (M.G.); wangxiping@nwsuaf.edu.cn (X.W.); Tel.: +86-29-87082613 (M.G.); +86-29-87082129 (X.W.)

† These authors contributed equally to this work.

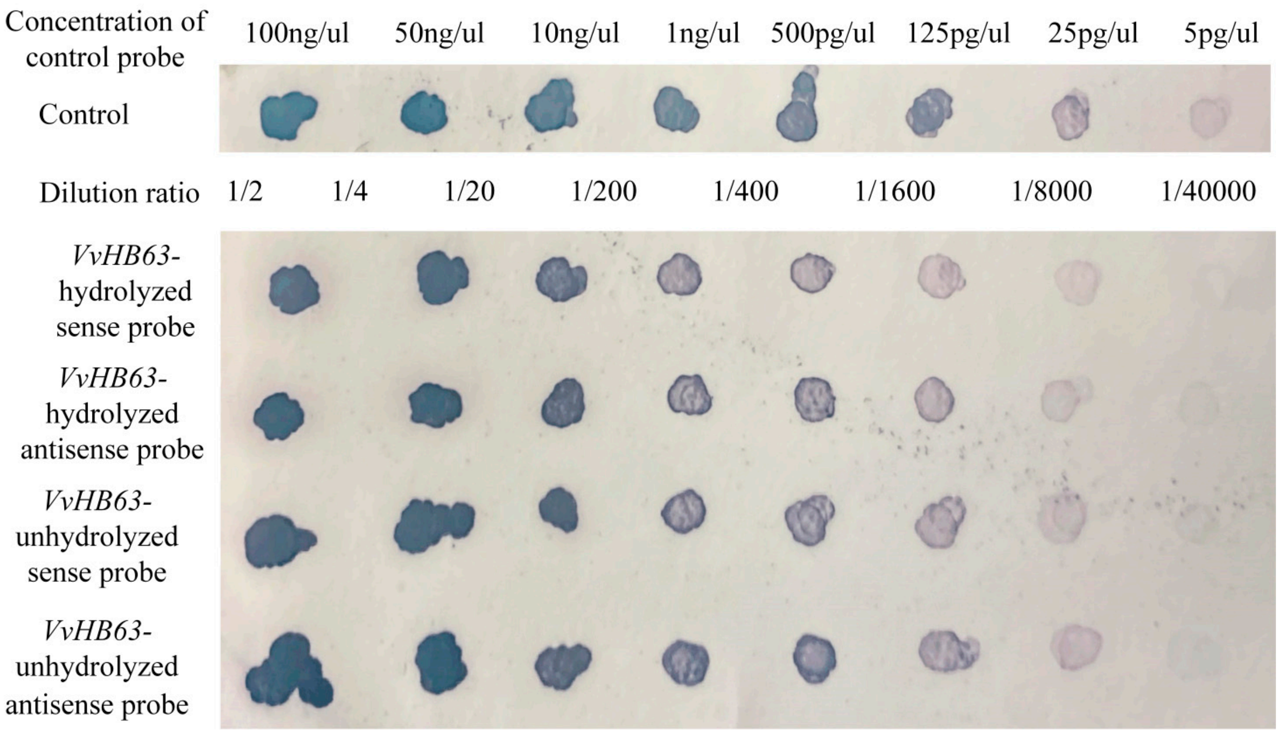

Figure S1. Semi-quantitative analysis of *VvTAU* probe by dot blot hybridization.

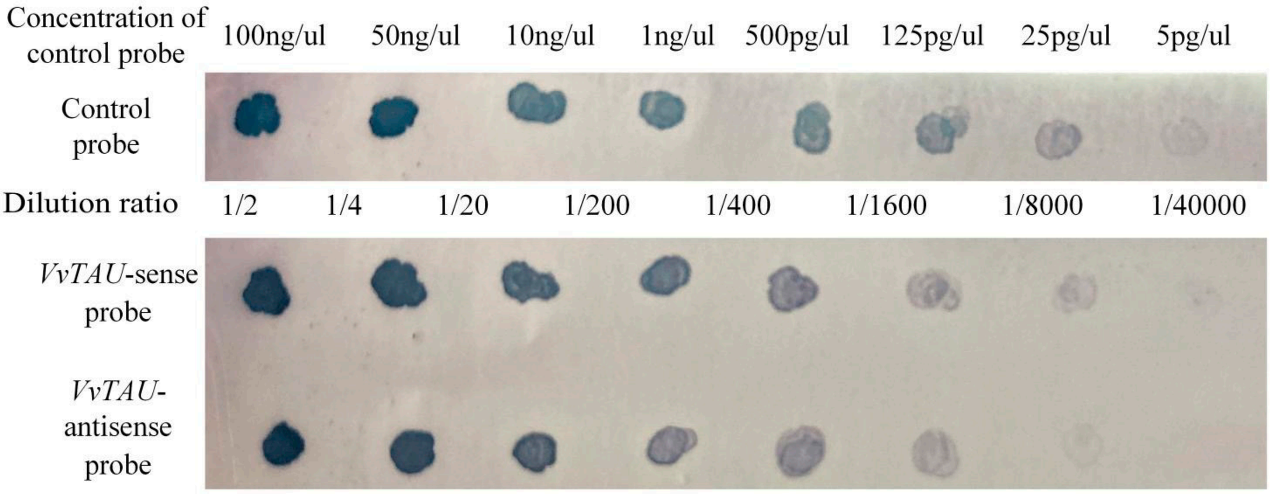

Figure S2. Semi-quantitative analysis of *VvHB63*probe by dot blot hybridization.

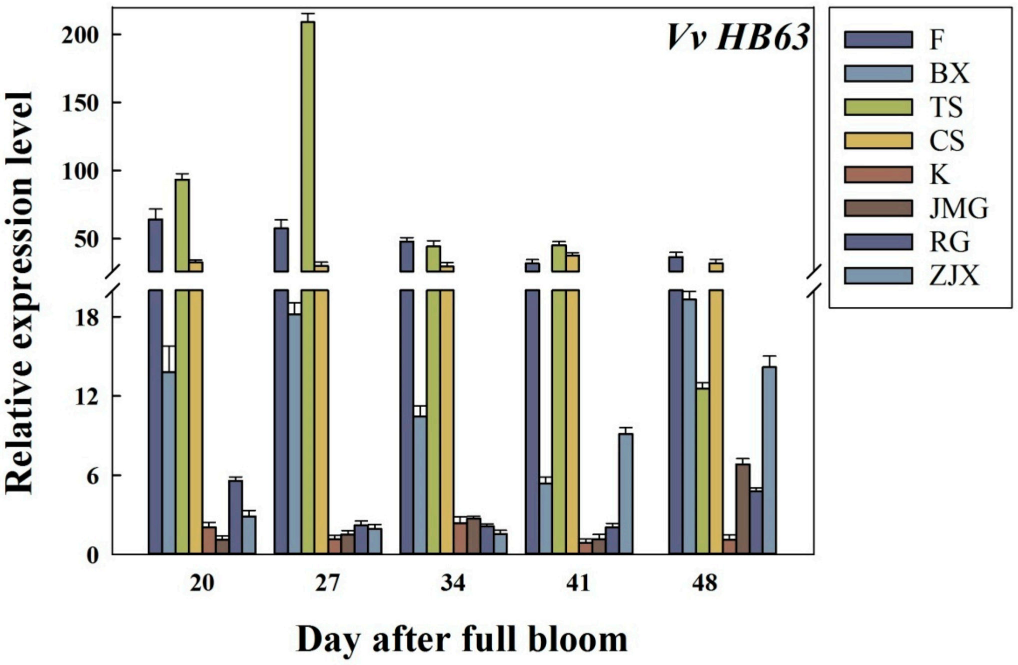

Figure S3. Expression patterns of grape *VvHB63* in ovules from eight grape cultivars. Seedless cultivars: Flame Seedless (F), Bi Xiang Seedless (BX), Thompson Seedless (TS), Crimson Seedless (CS), Seeded cultivars: Kyoho (K), Ju Mei Gui (JMG), Red Globe (RG), Zui Jin Xiang (ZJX). Using *VvGAPDH* and *VvEF1-a* genes followed by geometric averaging for determining a normalization factor.

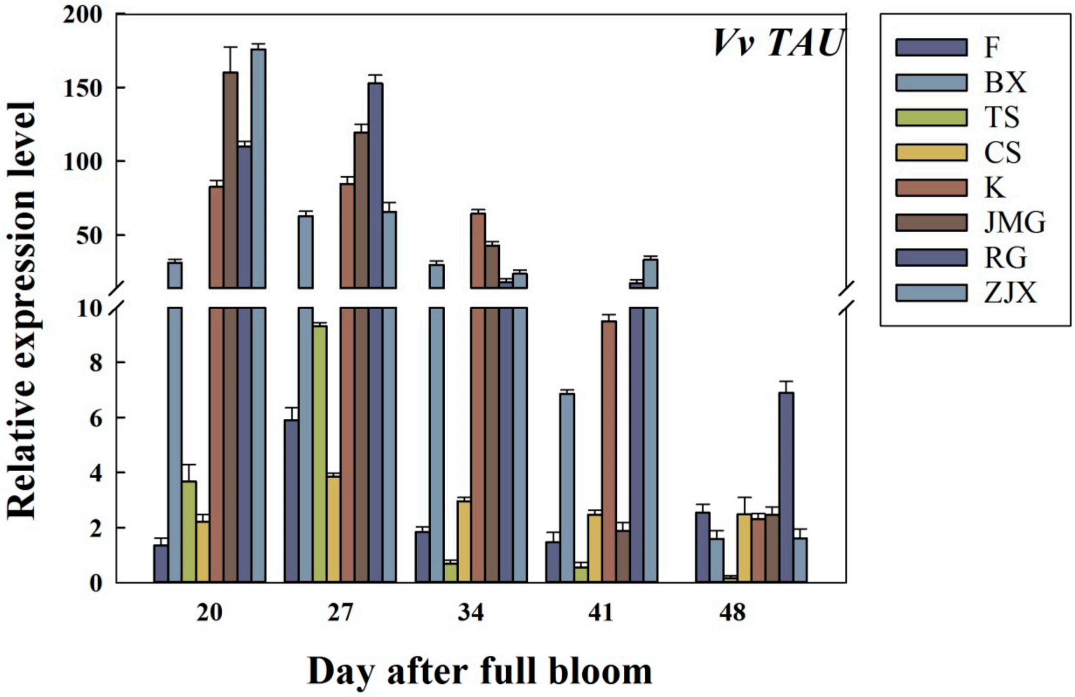

Figure S4. Expression patterns of grape *VvTAU* in ovules from eight grape cultivars. Seedless cultivars: Flame Seedless (F), Bi Xiang Seedless (BX), Thompson Seedless (TS), Crimson Seedless (CS), Seeded cultivars: Kyoho (K), Ju Mei Gui (JMG), Red Globe (RG), Zui Jin Xiang (ZJX). Using *VvGAPDH* and *VvEF1-a* genes followed by geometric averaging for determining a normalization factor.

Table S1: Reagent configuration method

Reagent name	component
2×carbonate buffer	32ul 1M $NaHCO_3$, 48ul 1M $NaCO_3$; 320ul DEPC-H_2O
10×PBS	1.3M NaCL; 70mM Na_2HPO_4; 30mM NaH_2PO_4; DEPC-H_2O
10×TBS	1M Tris-HCl pH7.5; 1.5M NaCl
20×SSC	3M NaCl; 300mM Na citrate
5×NTE	2.5M NaCl; 50mM Tris pH 8; 5mM EDTA; DEPC-H_2O
Buffer C	100mM Tris-HCl pH9.5; 50mM $MgCl_2$; 100mM NaCl
1% Blocking buffer	1% in 1×TBS
10x proteinase K buffer	100mM Tris pH8; 50mM EDTA
Acetic anhydride solution	3ml in 600ml 0.1M triethanolamine-HCl, pH 8 (11.142g, 600ml H_2O, 3ml 10M NaOH for the pH)
4% paraformaldehyde	4% in 1×PBS
10× in situ salts	3M NaCl ; 100mM Tris-HCl pH8; 100mM Na phosphate, pH 6.8; 50mM EDTA; DEPC H_2O to 10ml; Store at -20C
hybridization buffer	0.3 M NaCl; 10mM Tris-HCl pH 6.8; 10mM $NaHPO_4$ pH 6.8; 5mM EDTA; 50% formamide; 10% dextran sulfate; 1xDernhardts (0.02% each:Ficoll; PVP; BSA); 1mg/ml tRNA
